# Mitigating off-target effects in CRISPR/Cas9-mediated in vivo gene editing

**DOI:** 10.1007/s00109-020-01893-z

**Published:** 2020-03-20

**Authors:** Hua Alexander Han, Jeremy Kah Sheng Pang, Boon-Seng Soh

**Affiliations:** 1 Disease Modeling and Therapeutics Laboratory, A*STAR Institute of Molecular and Cell Biology, 61 Biopolis Drive Proteos, Singapore, 138673 Singapore; 2 Department of Biological Sciences, National University of Singapore, 14 Science Drive 4, Singapore, 117543 Singapore; 3 Key Laboratory for Major Obstetric Disease of Guangdong Province, The Third Affliated Hospital of Guangzhou Medical University, Guangzhou, 510150 China

**Keywords:** CRISPR/Cas9, In vivo, Gene therapy, Off-target effects, Clinical trials

## Abstract

The rapid advancement of genome editing technologies has opened up new possibilities in the field of medicine. Nuclease-based techniques such as the CRISPR/Cas9 system are now used to target genetically linked disorders that were previously hard-to-treat. The CRISPR/Cas9 gene editing approach wields several advantages over its contemporary editing systems, notably in the ease of component design, implementation and the option of multiplex genome editing. While results from the early phase clinical trials have been encouraging, the small patient population recruited into these trials hinders a conclusive assessment on the safety aspects of the CRISPR/Cas9 therapy. Potential safety concerns include the lack of fidelity in the CRISPR/Cas9 system which may lead to unintended DNA modifications at non-targeted gene loci. This review focuses modifications to the CRISPR/Cas9 components that can mitigate off-target effects in in vitro and preclinical models and its translatability to gene therapy in patient populations.

## Introduction

The possibility of editing the genome of an organism, inserting, deleting or modifying DNA sequences at targeted loci, holds great promise for the advancement of gene therapy in clinical populations. In 1990, the FDA approved the very first US-based gene therapy-related clinical trial in which 2 children afflicted with ADA-linked severe combined immuno-deficiency (ADA^−^SCID) were infused with T-cells that had a corrected copy of the *ADA* gene [1]. Both patients responded positively to the treatment, with tests showing an increased number of functional T-cells that were able to survive for over a year following the last infusion. However, the lymphocytic ADA enzyme levels in one patient did not differ significantly after treatment owing to the low efficiency of the transgene integration via homologous recombination (HR). In addition, it was difficult to determine the exact contributions of the gene therapy to the beneficial results given that the patients were continued on PEG-ADA treatments concurrently [1]. Consequentially, clinical trials over the subsequent 30 years have shifted towards using next-generation engineered endonuclease-based gene editing technologies with a focus on higher efficiency and specificity. These include, but are not limited to, zinc-finger nucleases (ZFNs), transcription activator-like effector nucleases (TALENs) and clustered regularly interspaced short palindromic repeats (CRISPR)/Cas9 endonucleases. Of the three, ZFN and TALEN are nucleases containing a customisable DNA-binding domain, which is commonly fused to a *Fok*I DNA-cleavage domain [2–4]. The CRISPR/Cas9 system consists of a DNA-cleaving endonuclease (Cas9) associated with a guide RNA that recognises and binds to the targeted sequences [5–7]. Regardless of the technique, the concept underlying gene editing with endonucleases is to induce a double-strand break (DSB) at targeted sites in the genomic DNA of the host cell, following which, DNA repair then proceeds via (1) the more predominant non-homologous end joining (NHEJ) that introduces a loss-of-function mutation through a reading frame shift or a premature stop codon or (2) a directed HR process that integrates transgene sequences into the genomic DNA through a supplied repair template [8]. These newer techniques demonstrated great therapeutic potential in ex vivo and in vivo preclinical models, which led to the approval of clinical trials in the USA that utilised these gene editing tools to ameliorate disease phenotypes [9–12].

**Fig. 1:**
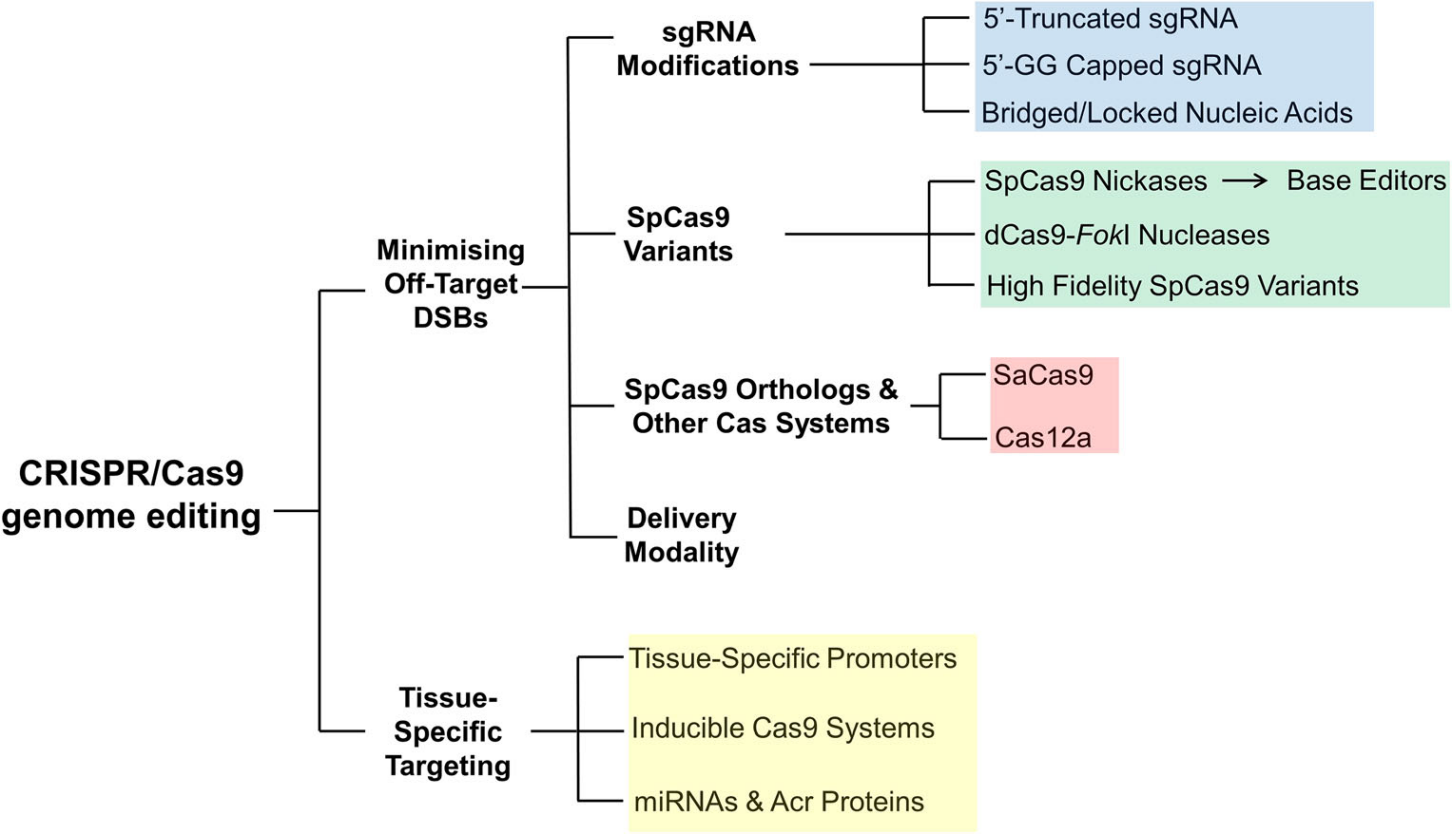
Graphical outline of the review article and overview of the mitigating techniques that can be applied to minimise OTEs in CRISPR/Cas9 genome editing

The results from the early phase clinical trials have so far been promising. However, given the small patient populations recruited into these trials, the safety of these gene therapies has yet to be fully evaluated. Specifically, potential off-target effects (OTEs) of these techniques have been highlighted in several studies demonstrating off-target ZFN and TALEN activity in in vitro and in vivo preclinical models [13–17]. Similarly, significant rates of OTEs had been reported in in vitro CRISPR/Cas9 edited human cell lines [18–22] and non-viable human embryos [23]. The CRISPR/Cas9 gene editing technique holds many advantages over ZFNs and TALENs in targeting disorders with a distinct genetic aetiology—the ease of designing the guide RNA sequences, the computational determination of OTEs based on genomic sequences with high similarity to the target locus (first-order sequence screens) [24–27] and the possibility of editing multiple genomic loci simultaneously [4]. Nonetheless, it remains prudent for clinical applications of CRISPR/Cas9 to implement constructs that can minimise OTEs. This current review focuses on modifications to the design of the CRISPR/Cas9 gene editing tool to mitigate OTEs and the subsequent delivery of CRISPR/Cas9 to the desired gene loci/cell populations in vivo to achieve effective gene editing clinically (Fig. 1).

## The CRISPR/Cas9 system

The CRISPR/Cas9 gene editing tool was developed from the endogenous prokaryotic immune system in which DNA sequences (CRISPR sequences or spacers) from invading viruses and phages incorporate into the bacteria or archaea as clustered interrupted repeats [28–30]. Together with the Cas endonuclease, these CRISPR sequences are used to detect and cleave DNA from subsequent infections from the same virus [31, 32]. The CRISPR/Cas system is divided into 2 classes, with class 1 consisting of types I, III and IV Cas proteins while class 2 consists types II, V and VI Cas proteins [33]. The system is further subdivided into 19 subtypes based on the type of Cas protein [33]. In the class I system, multiple Cas proteins form an effective complex to cleave foreign DNA sequences while only a single Cas protein is required to do so in the class II system [34]. The CRISPR/Cas9 system falls under the class 2 type II classification [33] and the system derived from the *Streptococcus pyogenes* bacteria is the most extensively studied of the Cas9 proteins [35].

**Fig. 2:**
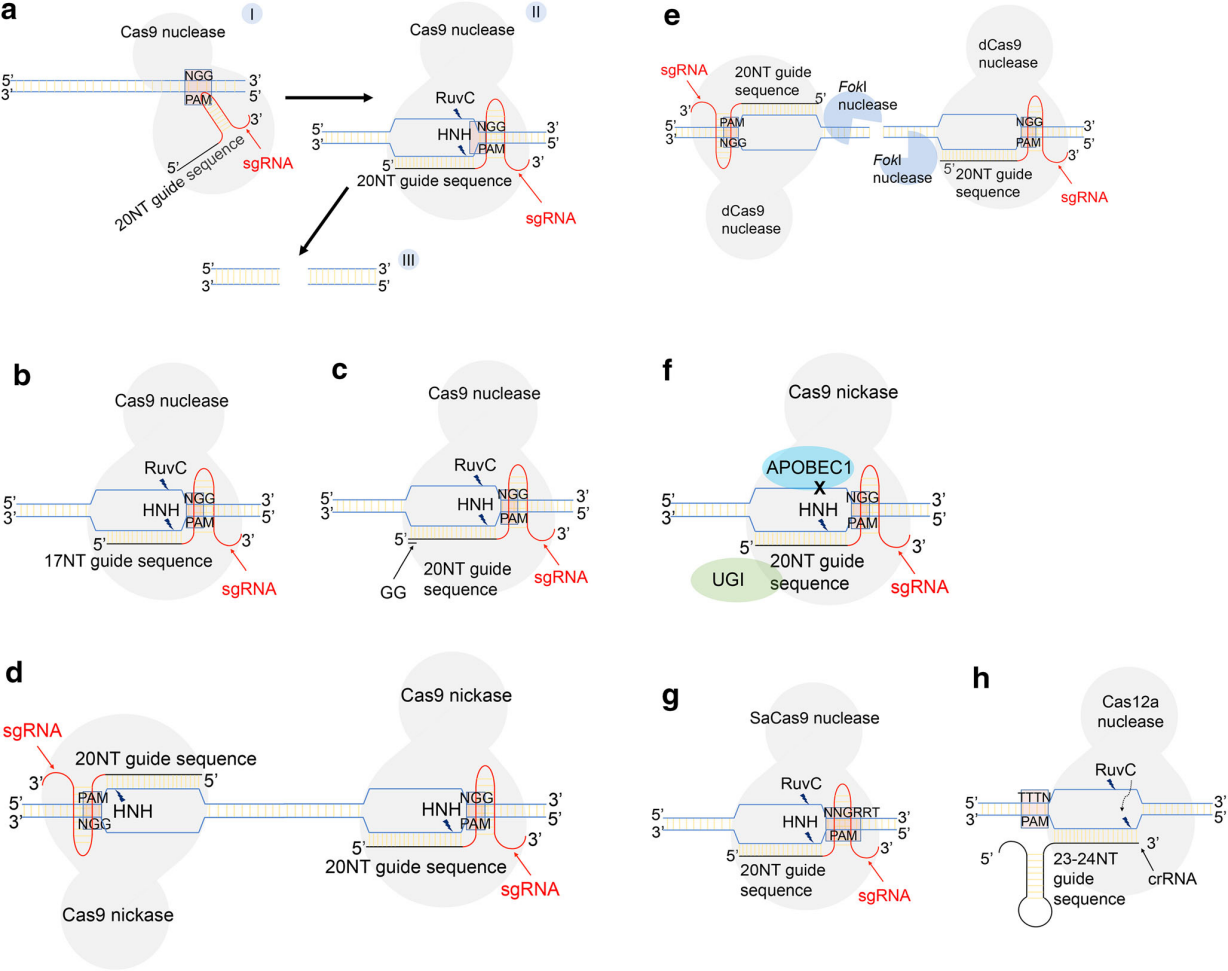
Stagewise schematic representations of target site recognition in CRISPR/Cas9-mediated genome editing with modifications to the sgRNA and Cas9 endonuclease to reduce OTEs. **a** The Cas9 endonuclease first scans the genomic DNA and binds to canonical PAM sequences (I). This induces a structural change in the sgRNA that allows the guide sequence to search and hybridise to complementary target sites upstream of the PAM (II). sgRNA-DNA hybridisation activates the Cas9 nuclease domains which then cleaves both strands of DNA (III). **b** sgRNA can be truncated at the 5′-end by 2–3 nucleotides or **c** modified at the 5′-end to contain 2 guanine nucleotides to improve the specificity of the guide sequence. **d** Cas9 nickase with only 1 active catalytic domain can be paired and **e** catalytically deactivated Cas9 fused to *Fok*I nuclease that requires dimerisation for nuclease activity can be used to minimise off-target indels (insertion/deletion). **f** Base editors that convert a single cytosine base to thymine without requiring DSBs are less promiscuous at off-target sites. **g** Cas9 orthologs from other bacteria such as SaCas9 and **h** other Cas nucleases such as Cas12a that recognises alternative PAMs can be used to target novel DNA sequences and improve specificity

Activation of the CRISPR system is initiated by the transcription of the repeat-spacer sequences into precursor CRISPR RNA (pre-crRNA) [36]. Together with RNase III and the Cas9 endonuclease, the pre-crRNA is processed into mature crRNA by a trans-activating crRNA (tracrRNA) hybridised at the 5′ end to the pre-crRNA [5, 37]. The mature crRNA-tracrRNA product then associates with the Cas9 endonuclease to form an RNA-endonuclease complex [5]. In addition to directing the processing of pre-crRNA, the tracrRNA is also critical in maintaining the Cas9 protein in the activated state [38]. The Cas9 protein searches the genomic DNA for the presence of protospacer adjacent motifs (PAMs) [39]—short DNA sequences recognised by the CRISPR/Cas system as foreign genomic material [40]. In *S. pyogenes*, the canonical 5′-NGG PAM sequence (Fig. 2a) lies downstream of the target site [5]. Once detected, *S. pyogenes* Cas9 (SpCas9) binds to the appropriate PAM sequence and relaxes the crRNA-tracrRNA structure to allow crRNA to scan for complementary DNA sequences [39]. In this way, the crRNA functions as a guide to bring Cas9 to the target site. Hybridisation of crRNA to the matching target sequence then induces a conformational change which activates the nuclease domains in Cas9 to cleave the target site [41].

The CRISPR/Cas9 system was modified for more efficient endeavours through the fusion of the 3′-end of crRNA to the 5′-end of tracrRNA to generate a single-guide RNA (sgRNA) that contained the target recognition domain of crRNA and a hairpin loop that mimicked endogenous interactions between tracrRNA and crRNA [5].

### Enhancing the fidelity of the CRISPR/Cas9 editing tool

Specificity of the CRISPR/Cas9 technique is a major concern in both preclinical and patient studies. Minimising OTEs while retaining/enhancing the rates of on-target activity of CRISPR/Cas9 is a challenge that involves DNA editing at the desired loci of the host genome, successful delivery of the CRISPR/Cas9 payload into the nucleus of the targeted cell populations, and in the case of in vivo gene editing, the correct tissues/organs. The techniques discussed in this section describe modifications to the sgRNA, Cas9 endonuclease and delivery modality of the CRISPR/Cas9 system with the common goal of eliminating DSBs at non-targeted gene loci (Fig. 2).

#### Designing and modifying the sgRNA

Selecting an appropriate sgRNA for the targeted DNA sequence is a crucial first step in avoiding OTEs. Many sgRNA design tools are available online that provide on-target and off-target predictions based on custom algorithms that may be species- and/or nuclease-specific. Factors such as the location of the cleave site within the gene, guanine content, counts and positions of mismatches between the sgRNA and protospacer sequence and non-canonical PAM sequences are duly taken into account in the models’ algorithms. A list of currently available sgRNA design web tools have been summarised by Cui et al. in a recent review article [42].

Conventional sgRNAs are designed to contain 20 nucleotides which are complementary to the target DNA sequence since longer sgRNA are found to be less effective [5, 6, 43–45]. However, Cas9 is able to bind to the target with a tolerance of up to 10 mismatches between the target sequence and the sgRNA while DNA cleavage can be detected at a small minority of loci with a mismatch of 3–5 base pairs [18, 20, 46]. To overcome this issue, the 5′-end of the sgRNA can be truncated to contain 17–18 nucleotides (Fig. 2b), hence increasing the sensitivity of sgRNA to mismatches without sacrificing on-target editing efficiency [47, 48]. On the contrary, truncation at the 3′-end of the sgRNA or by more than 3 nucleotides (16-nucleotide sgRNA) results in loss of on-target cleavage [18, 48, 49]. Truncating the 5′-end of the sgRNA is thought to increase the binding energy requirement between the sgRNA and DNA sequence, thus lowering the affinity of the sgRNA to off-target loci [48]. Inclusion of 2 guanine nucleotides at the 5′ end of the sgRNA also lowered the rate of OTEs significantly (Fig. 2c) but editing at on-target sites could also be reduce depending on the guide sequence [25, 50]. The underlying mechanism behind this change in specificity is unknown and intriguing given that an increase in GC content usually stabilises the hybridisation of RNA to DNA [51]. Finally, chemically modifying the central region of the 20-nucleotide guide sequence of the sgRNA through the inclusion of bridged or locked nucleic acids minimised OTEs [52]. The bridged/locked nucleic acids are thought to disrupt the stable state of the hybridised sgRNA-DNA complex at off-target sites, hence impeding the formation of DSBs.

#### Optimising the specificity of the Cas9 endonuclease

Cas9 protein from *S. pyogenes* contains the HNH and RuvC nuclease domains that cleave the DNA strands complementary and non-complementary to the guide sequence in sgRNA respectively [5]. Mutating either one of the catalytic residues of the nuclease domains (H840A in HNH and D10A in RuvC) transforms the Cas9 nuclease into a nickase that could only generate single-strand breaks (SSBs) with an overhang instead of blunt end cuts by wild-type Cas9 [5, 6]. It has been shown that using D10A-mutated Cas9 with a pair of sgRNA targeting sequences in proximity on opposite DNA strands (paired nickases, Fig. 2d) reduced OTEs by 100- to 1500-fold [50, 53–56]. Two SSBs, one on each DNA strand, generated in proximity to each other, essentially create a composite DSB. The decrease in OTEs is in part due to the low probability for off-target DSBs in this context [57, 58] while off-target SSBs are also repaired efficiently by local enzymes [59]. Cas9 with an inactivated RuvC domain has also been found to be more efficient than one with an inactivated HNH domain [6, 53].

Additionally, fusing the DNA catalytic domain of the *Fok*I endonuclease to a catalytically deactivated Cas9 protein (dCas9) also considerably reduces the OTEs of CRISPR/Cas9 gene editing [60–62]. The dCas9 protein serves as the DNA recognition domain while the *Fok*I nuclease domain induces a DSB following *Fok*I dimerisation at the correct spacing and orientation (Fig. 2e). Since the *Fok*I-dCas9 fusion protein requires a stricter target spacing of 15–25 base pairs for *Fok*I dimerisation than for paired Cas9 nickases, the resulting specificity of the *Fok*I-dCas9 protein is higher [60]. Despite the obligatory dimerisation of *Fok*I nucleases to generate a DSB, indels, most likely arising from *Fok*I monomers recruiting other monomers from solution, could still be detected at a lower frequency [62]. However, off-target indels induced by *Fok*I monomers can be reduced with the use of truncated sgRNAs. The enhanced specificity of the paired Cas9 nickases and *Fok*I-dCas9 protein is not without its drawbacks—the increased payload of the CRISPR/Cas9 components hinders effective delivery to the target cells/tissues.

Recent developments in CRISPR/Cas9 technology have also seen the emergence of base editors which can convert a single base into another without having to induce a DSB. A Cas9 nickase with a non-catalytic RuvC domain fused to APOBEC1, a cytidine deaminase enzyme, and uracil DNA glycosylase inhibitor (UGI) successfully converted a cytosine base to thymine (Fig. 2f) at the target site without having to create a DSB [63]. Similarly, the adenine base editor consisting of tRNA adenine deaminase and Cas9 nickase converts an adenine base to guanine [64]. These studies suggest the possibility of treating genetic disorders of point mutation origins at a lower risk of incurring OTEs. However, base editors can be limited by the narrow 5-nucleotide deamination window at the PAM-distal end of the protospacer as well as its inability to discriminate between specific cytosine residues within that window [63, 65]. In vitro assays using Digenome-seq and EndoV-seq have revealed higher genome-wide specificities of cytidine (9-fold) and adenine (2–20 fold) base editors when compared to Cas9 nuclease editing [66–68]. Recent studies adopting a more sensitive genome-wide detection of single-nucleotide variations confirmed that the cytidine base editors induced a 20-fold increase in single-nucleotide variations in mouse embryo when compared to controls while adenine base editors generated minimal single-nucleotide variations [69]. Since the nucleotide variations were predominantly cytosine to thymine conversions, these results further suggested that the cytidine base editor was potentially more promiscuous in on-target selection than the adenine base editor [69].

One of the prevailing theories hypothesise that specificity of the Cas9 endonuclease is governed by the binding energy threshold between the sgRNA-Cas9 complex and the DNA sequence. Wild-type sgRNA-Cas9 complexes contain more than optimal levels of energy for the binding of the complex to the target DNA locus; hence, the excess energy is able to accommodate off-target binding [48]. Through mutating the residues in the Cas9 protein which form hydrogen bonds with the DNA phosphate backbone (high-fidelity SpCas9-HF1) or substituting positively-charged amino acids with neutral alanine residues (enhanced specificity eSpCas9(1.1)), the Cas9 affinity for non-targeted sequences is diminished and the resulting Cas9 variants showed reduced OTEs [70, 71].

Recent studies on SpCas9-HF1 and eSpCas9(1.1) DNA binding assays, however, dispute the abovementioned hypothesis, showing that the binding affinities for on-target and off-target DNA sequences are comparable to wild-type SpCas9 [72]. Using single-molecule Förster resonance energy transfer (smFRET) techniques, the authors revealed that the HNH domains of SpCas9-HF1 and eSpCas9(1.1) remained in a catalytically inactive state when the nucleases were bound to off-targets [72]. In this way, alanine substitutions in these high-fidelity Cas9 proteins increase the threshold required for the HNH domain to switch to a conformationally active state [41, 73]. Based on these observations, a new hyper-accurate SpCas9 variant (HypaCas9) was designed to contain alanine substitutions in the non-catalytic REC3 domain, which restricted a downstream conformation change to an activated HNH domain when Cas9 is bound to off-targets [72]. When compared to SpCas9-HF1 and eSpCas9(1.1), HypaCas9 exhibited even greater specificity than the other 2 variants. GUIDE-seq assay revealed that with the sgRNA targeting *VEGFA* site 2, 18 off-target sites were detected for HypaCas9 while 19 and 24 off-target sites were detected for eSpCas9(1.1) and SpCas9-HF1, respectively [72]. Other high-fidelity variants of SpCas9 nucleases such as evoCas9 and xCas9 were developed through library screens of REC3-mutated SpCas9 and phage-assisted continuous evolution respectively [74, 75]. Similar to SpCas9-HF1, eSpCas9(1.1) and HypaCas9, evoCas9 and xCas9 displayed substantial reductions in off-target editing compared to wild-type SpCas9 while having near wild-type on-target efficiency. Under the GUIDE-seq analysis, evoCas9 showed a 98.7% reduction in genome-wide off-target indels compared to wild-type SpCas9 while at known promiscuous sites such as *HEK* site 4 and *VEGFA*, xCas9 showed a 4.2- to 9.4-fold reduction in off-target/on-target ratios compared to wild-type SpCas9 [74, 75].

One of the requirements for a Cas9-induced DSB is the presence of proximal PAM sites to the targeted DNA sequences [5]. The precision of the cleave site may therefore be restricted by the location of PAMs within the vicinity of the targeted sequence. To circumvent this requirement, several measures can be adopted including (1) engineering SpCas9 to recognise alternative PAM sequences, (2) Cas9 orthologs from other bacteria such as *Staphylococcus aureus* (SaCas9), and (3) other types of CRISPR/Cas system such as the type V Cas12a (formerly Cpf1) that possess different PAM preferences can be used.

In one study, residues in the PAM-interacting domain of SpCas9 that contact the nucleotides of the PAM sequence were mutated under different permutations and through a bacterial selection system, the resulting SpCas9 variants were segregated based on their ability to discriminate between the canonical 5′-NGG and non-canonical 5′-NGA PAM [76]. Previous reports have documented that wild-type SpCas9 is able to induce DSBs at non-canonical PAMs at a lower frequency [46, 53]. One of the variants with a D1135E mutation were able to demonstrate a greater discernment against non-canonical PAMs when tested in human cell lines while maintaining comparable activity at targeted sequences with canonical PAMs to wild-type SpCas9 [76]. The augmented specificity of the D1135E mutant also extended to off-target sites that do not contain non-canonical PAMs. In the same study, 2 SpCas9 variants, VRQR and VRER, with mutations at positions 1135, 1218, 1335 and 1337, were found to possess an enhanced affinity for 5′-NGA and 5′-NGCG PAM sites over wild-type SpCas9, respectively. The availability of SpCas9 variants with an expanded range of PAM preferences could therefore grant the accessibility to previously hard-to-target genes as well as limit the frequency of OTEs owing to the less commonly occurring PAMs [75].

Alternatively, Cas9 orthologs from other species such as SaCas9 (Fig. 2g) with a longer PAM requirement of 5′-NNGRRT (R = A or G) can be used to mediate gene editing in vivo with a similar efficiency and specificity to SpCas9 [76, 77]. The advantage of a smaller-sized SaCas9 protein relieves the issue of packaging into viral delivery systems with a limited payload and the requirement for a less frequently occurring PAM innately lowers the probability of OTEs [77]. The SaCas9 protein can also be modified to accept laxer PAM sequences such as the KKH-SaCas9 variant with a 5′-NNNRRT PAM requirement; however, this can translate to an increased likelihood of OTEs compared to native SaCas9 [78].

Finally, the Cas12a nuclease from *Acidaminococcus sp. BV3L6* and *Lachnospiraceae bacterium ND2006* (Fig. 2h) offers an attractive and higher fidelity option to CRISPR/Cas9-mediated genome editing of human cell lines [79, 80]. In comparison to CRISPR/Cas9 systems, the type V CRISPR/Cas12a system is able to process pre-crRNA into mature crRNA without a tracrRNA, hence reducing the size of the plasmid constructs [79]. In a recent study, a single, carefully designed pre-crRNA had been shown to be sufficient in driving simultaneous multiplex gene editing in HEK293T cells and mouse neurons in vivo [81]. The Cas12a protein recognises a T-rich (5′-TTTN) PAM sequence instead of the 5′-NGG PAM by its Cas9 counterpart, thus offering a greater precision in targeting novel gene loci [79]. This has been reflected in a study that utilised the Cas12a nuclease to correct mutations in the dystrophin gene (*DMD*) that causes Duchenne muscular dystrophy (DMD) [82]. Additionally, Cas12a-induced DSBs generate a 5′ overhang of 4–5 nucleotides that can facilitate gene insertion via the NHEJ repair pathway as opposed to the blunt end cuts generated by Cas9 [5, 79, 83]. Like the SpCas9 and SaCas9 endonucleases, Cas12a can be engineered to improve on-target activity and reduce off-target mutations in human cell line editing [84].

In summary, Table 1 provides a consolidated overview of the aforementioned engineered Cas9 variants, Cas9 orthologs and Cas nuclease from other systems.

**Table 1:** List of natural-occurring and engineered Cas endonucleases.

Cas Protein	Modifications	Remarks on OTEs	Reference
SpCas9 nickase	D10A mutation to deactivate RuvC nuclease domain of SpCas9	Reduction of OTE by 100 to 1500 folds compared to wild-type SpCas9 while maintaining similar on-target efficiency	[46, 55]
dSpCas9-*Fok*I	Fusion of deactivated SpCas9 protein to catalytic domain of *Fok*I endonuclease	Specificity of dSpCas9-*Fok*I assessed at *CLTA*, *EMX* and *VEGF* target loci with 2 sgRNAs targeting each locus. No detectable OTEs at 11 known off-target sites analysed. On-target:off-target ratios were >140-fold higher in dSpCas9-*Fok*I induced mutations than wild-type SpCas9. Deeper interrogation of *VEGF* off-target site 1 revealed off-target cleavage by dSpCas9-*Fok*I and SpCas9 nickase. On:off ratio was 1.3 to >10-folds higher in dSpCas9-*Fok*I than SpCas9 nickase.	[62]
C˃T Base editor	Fusion of SpCas9 HNH nickase to rat APOBEC1 cytidine deaminase enzyme and UGI	Comparing specificities of wild-type SpCas9 to C>T base editors at 7 target loci: *EMX*1, *FANCF*, *HEK*2-4, *RNF*2 & *HBB*, an off-target effect index was used for each of the 7 sgRNAs. The index was defined as cumulative mutation frequencies at validated off-target sites divided by on-target mutation frequencies. Base editor indices ranged from 0 – 1.7 while indices for SpCas9 ranged from 0.0049 – 2.8. Corresponding indices were always higher for SpCas9. Extension or truncation of sgRNAs improved specificity of base editor at most sites.	[68]
A>G Base editor	Fusion of catalytically deactivated SpCas9 to *E. Coli* adenine deaminase	Specificities of the A>G base editor were analysed by EndoV-seq at 8 target loci: *HEK*2, *EMX*1, *HBG*, *HBB-28* (A>G) mutant allele, *FANCF*, *RNF*2, *VEGFA*3, *DMD*, using 7 sgRNAs. *HBG* sgRNA can target *HBG*1 and *HBG*2. At a genome-wide cutoff cleavage score >2.5, total off-target sites ranged from 2-19 for A>G base editor, 0-31 for C>T base editor and 7-320 for SpCas9. At the *VEGFA*3 target locus (cutoff score>2.5) the total off-target sites was 19, 2, and 231 for A>G, C>T base editors and SpCas9 respectively.	[70]
SpCas9-HF1	Point mutations in SpCas9 at N497A, R661A, Q695A, Q926A	The GUIDE-seq technique was used to evaluate off-target effects at *EMX*1, *FANCF*, *RUNX*1 and *ZSCAN*2 loci with 8 sgRNAs targeting these sites. OTEs of SpCas9 were detected at all loci except for the *FANCF*-4 site. The number of off-target sites ranged from 2-25. No OTEs of SpCas9-HF1 were detected except on 1 site at the *FANCF*-2 locus. At *VEGFA* site 2 and 3, SpCas9-HF1 induced OTEs at 21 and 1 site respectively while SpCas9 induced OTEs at 144 and 32 sites respectively. However, the on-target:off-target ratios at *VEGFA* site 3 were comparable for SpCas9 and SpCas9-HF1.	[72]
eSpCas9(1.1)	Point mutations in SpCas9 at K848A, K1003A and R1060A	Specificity of eSpCas9(1.1) was examined at the *EMX*1(1), *VEGFA*(1) & *VEGFA*(5) loci. eSpCas9(1.1) induced OTEs only at 1 out of the 24 predicted off-target site (*VEGFA*(1)). Wild-type SpCas9 with 20nt sgRNA induced OTEs in 5, 4 & 11 sites at the *EMX*1(1), *VEGFA*(5) & *VEGFA*(1) loci respectively. Wild-type SpCas9 with truncated sgRNA (17nt for *VEGFA* and 18nt for *EMX*1) induced OTEs in 3 & 4 sites at the *VEGFA*(5) & *VEGFA*(1) loci respectively but increased off-target activity 5 other sites compared to full length sgRNA + SpCas9. Using BLESS analysis to evaluate specificity at *EMX*1(1) and *VEGFA*(1), eSpCas9(1.1) reduced DSB scores at off-target sites compared to wild-type SpCas9.	[73]
HypaCas9	Point mutations in SpCas9 at N692A, M694A, Q695A, H698A	GUIDE-seq analysis was performed to determine specificity at 6 loci: *FANCF* site 2, 6, *DNMT1* site 3, 4 & *VEGFA* site 2, 3 using 6 sgRNAs. HypaCas9 induced OTEs in 1, 1, 18 & 1 site at the *FANCF*2, *DNMT1* site 3, *VEGFA* site 2 & 3 loci respectively. In comparison, wild-type SpCas9 induced OTEs in all sampled loci except for *DNMT1* site 3 (10 for *FANCF*2, 34 for *FANCF*6, 8 for *DNMT1* site 4, 134 for *VEGFA* site 2, 12 for *VEGFA* site 3). HypaCas9 also induced lower OTEs than SpCas9-HF1 and eSpCas9(1.1) at the *VEGFA* site 2 locus (24, 19 & 18 sites respectively)	[74]
evoCas9	Point mutations in REC3 domain of SpCas9 at M495V, Y515N, K526E, R661Q	GUIDE-seq analysis was performed to determined the specificity of evoCas9 at 8 loci: *CCR5*, *CXCR4*, *FANCF*2, *HEK* site 4, *EMX*1, *PD1*, *VEGFA*2 & 3. evoCas9 induced fewer off-target events (by 98.7%) at the sampled loci compared to wild-type SpCas9. At the 12 off-target sites cleaved by evoCas9 (including 9 for *VEGFA*2, 1 for *VEGFA*3, *FANCF*2, *CCR5* each), the on-target:off-target ratio was significantly higher in evoCas9 compared to wild-type SpCas9. Targeted deep sequencing of the *VEGFA*2 locus also revealed a significantly higher on-target:off-target ratio.	[76]
xCas9(3.7)	Multiple point mutations in SpCas9	Guide-seq data revealed that the number of off-target events induced by xCas9(3.7) were significantly lower than wild-type SpCas9 at all of the 8 target loci: *EMX*1, *HEK* site 1-4 in HEK293T cells and *EMX*1 & *VEGFA* in U2OS cells. The off-target:on-target ratio for the targeted loci ranged from 0.14 – 9.4 for SpCas9 while the range for xCas9(3.7) was <0.001 – 1.0. In particular, the ratio for the *VEGFA* locus for SpCas9 was 2.0 and 0.48 for xCas9(3.7).	[77]
VRQR-SpCas9	Point mutations in PAM-interacting domain of SpCas9 at D1135V, G1218R, R1335Q, T1337R	GUIDE-seq analysis was performed at 8 targeted loci: *EMX*1 site 4, *FANCF* site 1, 3, 4, *RUNX*1 site 1& 3, *VEGFA* site 1, *ZNF629*. The number of off-target cleavage sites induced by VRQR-SpCas9 were similar to wild-type SpCas9 (PAM=5’-NGA)	[78]
VRER-SpCas9	Point mutations in PAM-interacting domain of SpCas9 at D1135V, G1218R, R1335E, T1337R	GUIDE-seq analysis was performed at 5 targeted loci: *FANCF* site 3 & 4, *RUNX*1 site 1, *VEGFA* site 1 & 2. The number of off-target cleavage sites induced by VRER-SpCas9 were slightly lower than wild-type SpCas9 (PAM=5’-NGCG)	[78]
SaCas9	Cas9 nuclease from *S. aureus*	GUIDE-seq analysis was used to evaluate SaCas9 specificity at the *VEGFA* site 3 locus using a 20nt-sgRNA. DSBs were induced at 8 identified off-target sites by SaCas9 and SpCas9. On-target:off-target ratio was higher for SaCas9 compared to SpCas9	[87]
Cas12a	Cas12a nuclease from *Acidaminococcus sp. BV3L6* and *Lachnospiraceae bacterium ND2006*	Digenome-seq was used to compared genome-wide specificity of Cas12a to SpCas9 with 2 Cas9 sgRNAs that target the 2 Cas12a sites at the *DNMT1* locus. The off-target:on-target ratios for AsCas12a were 0.267 & 0.024 and 0.005 & 0.012 for LbCas12a. In contrast, the ratio for SpCas9 was >2.0.	[88]

#### Modality of the CRISPR/Cas9 system during delivery

The CRISPR/Cas9 system consisting of the sgRNA and Cas9 nuclease can be delivered in several forms into the host cell for genome editing. The cDNA of the Cas9 nuclease as well as the sgRNA can be expressed in a single plasmid [44] or separated into 2 plasmids to be delivered concurrently into the host cell [7]. While the design and cloning of the expression cassette is relatively non-complicated, the use of plasmid DNA can potentially lead to the integration of unwanted DNA sequences into the host genome [87]. Prolonged expression of the sgRNA and Cas9 nuclease due to the persistence of plasmid DNA can also increase the potential of OTEs [88]. In addition, introduction of foreign DNA can trigger cellular immune responses [89]. One study proposed an engineered plasmid expression cassette to contain 2 sgRNAs, one targeting the gene of interest and the other targeting the Cas9 itself [90]. Designed in this manner, the Cas9 transgene would be cleaved simultaneously with the gene of interest, hence reducing the duration of Cas9 expression. As expected, the Cas9 nuclease expression level was found to be diminished by post-treatment day 2 while measured OTEs were significantly reduced correspondingly [90].

The Cas9 endonuclease can be delivered in the form of mRNA into the host cell. Switching to Cas9 mRNA prevents the integration of plasmid DNA into the host genome and reduces the propensity for OTEs by reducing the exposure time to Cas9 nuclease [91, 92]. Delivering the Cas9 nuclease as a mRNA precursor also hastens the onset of CRISPR/Cas9-mediated gene editing since the process bypasses the transcription of Cas9 cDNA [91].

Finally, Cas9 can be delivered as a protein complexed with the mature sgRNA to form a ribonucleoprotein (RNP) assembly [91, 93–95]. Cas9 RNPs are able to generate genomic on-target DSBs immediately after delivery and are rapidly broken down by endogenous proteases [91, 96]. By titrating the concentration of the Cas9 RNPs to an optimal level and limiting the window of exposure to the RNPs, OTEs in the edited cells can be substantially reduced by 2.2 to 19 folds [91, 93, 95, 96]. Similar to using Cas9 mRNA, cellular toxicity associated with exogenous DNA and the possibility of foreign DNA integration can be avoided with Cas9 RNP [97].

## DNA repair pathways

Resolution of DSBs generated by CRISPR/Cas9-mediated gene editing can proceed via 2 pathways: NHEJ and homology-directed repair (HDR). In mammalian cells, NHEJ is the predominant repair pathway that competes with the less efficient HDR pathway [98, 99]. NHEJ is an error-prone but efficient pathway that creates indels, leading to frameshift mutations and eventually functional gene knockouts [8, 100]. Using 2 DSBs simultaneously, the exon-coding sequence or regulatory element of genes can also be deleted and the DNA cleavage rectified via NHEJ [101–103]. In tumourigenesis, where mutated oncogenes such as *KRAS* and *EGFR* initiate the proliferation and survival of cancerous cells, the CRISPR/Cas9 system can generate DSBs within the exon-coding regions of the mutated alleles, which is then repaired via NHEJ to form indels that disrupt the oncogenes and inhibit the survival and tumourigenicity of the mutant cells [104, 105]. This CRISPR/Cas9-mediated anti-tumourigenic effect has also been replicated in in vivo xenograft models of cancer, with additional beneficial effects of abrogating angiogenesis and metastasis of the cancerous cells [105–108].

However, gene therapy can also require the correction of a defective gene through the repair of a point mutation or the precise integration of a functional gene copy into the genome of the target cells. This can be accomplished through the HDR pathway with an exogenously supplied donor repair template, often in the form of single-stranded oligodeoxynucleotides (ssODNs) for point mutation corrections or plasmid DNA templates for entire transgenes [6, 7, 109, 110]. Successful HDR-mediated gene editing is determined by the length of the repair template and flanking sequences that are homologous to either side of the DSB. Larger repair templates require longer homology arms with a minimum of 400 base pairs for efficient editing [111, 112]. In contrast, ssODNs do not require long homology arms to correct point mutations [100]. Improvement in HDR efficiency can also be achieved through using asymmetrical donor template [113, 114].

To bias the DNA repair mechanisms towards HDR-mediated pathways, studies have reported using small-molecule inhibitors such as Scr7, shRNA and proteins that target DNA ligase IV, an important enzyme in the NHEJ pathway [99, 115] and RS-1, an HDR enhancer that exerts its effect through stimulating the human HR RAD51 protein [116]. Others have also synchronised the delivery of the sgRNA and Cas9 nuclease to the late G2 phase of the cell cycle or fusing the Cas9 nuclease to the human Geminin protein to capture the active time window of HDR pathways during late S and G2 phases of the cell cycle [117, 118]. Additionally, the donor template can be tethered to the Cas9 nuclease to improve HDR efficiency [119, 120].

However, despite these innovative executions to favour the HDR repair pathway, precise gene editing remains a challenge in post-mitotic cell populations such as neurons since HDR is confined to the late S and G2 phases of the cell cycle. The emergence of base editors thus presents an attractive alternative for inducing single nucleotide mutation in these cell populations since the repair mechanism does not depend on HR pathways [63, 64, 121]. Likewise, other studies have hijacked the NHEJ repair machinery in developing a homology-independent targeted integration (HITI) which demonstrated more robust knock-in efficiencies for the *MERTK* gene in postnatal mouse neurons as compared to the traditional HDR method [122]. The transgene is integrated in a specified orientation since the sgRNA/SpCas9 complex is designed to cleave off the donor DNA if it is inserted in the reverse direction [123].

## Targeting specific tissue/cell populations in vivo

In most mammalian systems, there is a very low likelihood of a homogenous cellular composition within any given tissue or organ. This presents a considerable barrier when genome editing is to be restricted to a selected population of cells in the tissue. The expressions of Cas9 endonuclease and sgRNA are typically driven by RNA polymerase II and III promoters respectively [124]. Viral RNA polymerase II promoters such as cytomegalovirus (CMV) and simian virus 40 (SV40) and eukaryotic promoters such human elongation factor 1 alpha (EF1α) and chicken β-actin (CBA) RNA polymerase II promoters are commonly used and are constitutively active [125, 126]. Viral RNA polymerase II promoters are preferred over eukaryotic promoters because viral promoters induce a higher level of transcription [127]. Expression of the sgRNA is usually regulated by an RNA polymerase III promoter such as the human U6 promoter which transcribes genes that encode for small RNA sequences in eukaryotes [124, 128]. This is because sgRNA expressed under the RNA polymerase II promoter is non-functional when unique sequences like the 5′-cap and 3′-polyadenylation tail are included in the transcript [124].

### Choice of promoter regions and inducible CRISPR/Cas9 systems

Since most of the frequently-used RNA polymerase II and III promoters in CRISPR/Cas9 genome editing are ubiquitously expressed in many cell types, they are not feasible when cell-type specific editing is required [125]. Hence, switching over to tissue-specific promoters to drive the targeted expression of Cas9 endonuclease should be considered when designing the vector plasmid. For example, the promoters for hsynapsin and cardiac troponin T (cTnT) are respective neuron- and cardiomyocyte-specific RNA polymerase II promoters that drive gene expression downstream [129–131]. A selected list of cell-specific promoters is available in Table 2. On a precautionary note, many native promoters, once thought to dictate gene expression in a specific cell type, are also active in other cell populations. Glial fibrillary acidic protein (GFAP) is predominantly expressed in astrocytes [132], but it can also be found in human keratinocytes and testicular Leydig cells [133, 134].

**Table 2:** List of cell-type-specific promoters for in vivo genome editing

Promoter	Tissue/cell type	Reference
hSYN1	Neuron	[135]
Aldh1L1	Astrocyte	[136]
cTnT α-MHC	Cardiomyocyte	[137] [138]
SP-C	Pulmonary alveolar type II cell	[139]
MUC2	Intestinal goblet cell	[140]
Ksp-cadherin	Renal tubular epithelial cell	[141]
Albumin	Hepatocyte	[142]
HSA	Skeletal muscle	[143]
Insulin	Pancreatic beta cell	[144]
Rhodopsin Cone-opsin	Retinal rod cell Retinal cone cell	[145]

The integration of an inducible promoter upstream of the tissue-specific promoter can impose spatial and temporal control over Cas9 expression, culminating in lower OTE frequencies given that one of the determinants of OTE rates is the length of time the genome is exposed to the nuclease [47, 91]. Studies have used the TRE3G (Tet-On-3G) and TRE2 Tet-On promoters to activate Cas9 proteins in an animal model and human immortalised cell lines respectively under the control of administered doxycycline [146, 147]. Lower OTEs were observed when compared to constitutively active Cas9 controls and embryonic lethality was avoided in the mouse model when the *APC* and *TRP53* tumour suppressing genes were targeted only during young adulthood [146]. This design could be adapted to regulate sgRNA expression and to restrict CRISPR/Cas9 editing to selected cell types in vivo within a designated time period or developmental stage [148, 149].

Other inducible systems aimed at regulating the sgRNA and Cas9 time window of activity have been described in previous literature. These include the use of ligand-activated aptazyme-embedded guide RNA [150], allosteric modulation of ligand-sensitive Cas9 [151], and conditionally-activated Cas9-intein and Cas9-destabilised domain fusion proteins [152–154]. Alternatively, temperature-sensitive Cas9 variants that operate within clinically tolerable ranges can be considered as a viable inducible option that avoids the potential toxicity of small-molecule ligands [155].

### microRNAs and anti-CRISPR proteins

More recently, studies have harnessed the cell-type specificity of microRNAs (miRNA) and anti-CRISPR (Acr) proteins in refining the fidelity of CRISPR/Cas9-mediated editing [156]. Acr proteins are derived from prophages that evolve to escape bacterial immunity by inhibiting the CRISPR/Cas defence mechanism [157]. Among the Acr proteins, AcrIIA2 and AcrIIA4 isolated from the *Listeria monocytogenes* prophage specifically target the SpCas9 nuclease, with AcrIIA4 in particular interfering with DNA recognition at the PAM-interacting domain and hindering the RuvC catalytic domain [157–160]. On the other hand, miRNAs are short, non-coding RNA sequences that regulate protein expression by targeting their mRNA for degradation or inhibiting the translation pathway [161].

Taking advantage of both systems, miRNA response elements (MREs) can be inserted into the 5′- or 3′-UTR of AcrIIA4 transgenes so that Acr protein expression levels can be regulated by cell-specific miRNAs [162–164]. Using a cardiomyocyte-specific miR-1, the expression of AcrIIA4 is repressed, allowing the sgRNA-SpCas9 complex to bind and cleave the target DNA sequence within the genome of cardiomyocytes. Conversely, since miR-1 is not present in off-target cells, AcrIIA4 is highly expressed and thereby inhibits sgRNA-SpCas9 binding to DNA [164]. This technique was replicated with miR-122 which demonstrated specificity in hepatocytes.

Cell-type-specific miRNA-mediated genome editing can also be achieved through a simplified protocol. In one study, Wang and colleagues designed a unique pre-sgRNA construct that consisted of an sgRNA flanked by an MRE on each side under the influence of the CAGGS RNA polymerase II promoter [165]. Due to the presence of a 5′-cap and 3′-polyadenylation tail, the pre-sgRNA remained non-functional until the regulatory elements were cleaved off by endo- or exogenous cell-type specific miRNA. CRISPR/Cas9-mediated genome editing can therefore be restricted to targeted cell types that express the complementary miRNAs to the MREs [165].

Despite the improved specificity conferred by the inducible and the Acr/miRNA-mediated CRISPR/Cas9 gene editing systems, several issues have to be addressed before these techniques can be translatable clinically. Small-molecule ligands can induce cytotoxicity and immunogenic responses [166] while the leakiness of Tet-On/Tet-Off systems limits its use in patient populations [146, 148]. The delivery efficiency of a large payload that includes the Acr and Cas9 transgenes, sgRNA and the repair template to the same cell may be low, while the persistence of foreign Acr proteins beyond a few days raises significant concerns.

### Targeted delivery of the CRISPR/Cas9 system in vivo

In vivo gene delivery approaches can be broadly divided into viral and synthetic non-viral vectors that are either locally or systemically administered. Viral vectors are able to mediate efficient gene transfer to the target cells but they carry the potential to elicit immunogenic and cytotoxic responses [167–169]. Moreover, packaging the CRISPR/Cas9 components into the virus can be a challenge. The cargo size limit for the popular adeno-associated virus (AAV) vector is around 4.7 k bp [170] while the size of the commonly-used SpCas9 is around 4.2 k bp alone [171]. While it is possible to concomitantly deliver SpCas9 and the sgRNA in the same vector, there is little room available for the inclusion of regulatory elements and donor repair templates [171, 172]. To bypass this issue, SpCas9 can be truncated [173] or split into 2 domains delivered separately [174]. SpCas9 can also be substituted by smaller Cas9 orthologs such as the ~3.2 k bp SaCas9 [77, 85].

In comparison, non-viral synthetic vectors carry a lower risk of initiating immunogenic events and lack the viral machinery to integrate exogenous DNA material into the host genome [175]. The payload capacity can also be expanded to contain the sgRNA, Cas9 nuclease and donor template within a single vector with ease [176] or to deliver the CRISPR/Cas9 components as an RNP [95, 177]. Furthermore, synthetic vectors are easy to manufacture in a large scale [178]. However, the main drawback of a non-viral vector system is that the delivery efficiency is often several folds lower than viral-mediated gene delivery [175, 179]. Despite the growing interest in synthetic vectors, the majority of the clinical trials involving gene therapy still use locally administered viral vectors because of the more robust delivery efficiency while restricting systemic off-targets [180].

Non-viral delivery of CRISPR/Cas9 components encapsulates the contents within a lipid, polymer or inorganic carrier or by conjugating the sgRNA/Cas9 nuclease with peptide sequences. Using a synthetic vector offers the option of delivering the sgRNA and Cas9 nuclease as an RNP which reduces the probability of OTEs by lowering the exposure time to the sgRNA/Cas9 nuclease [91, 95, 176]. Furthermore, synthetic vectors can be engineered to target specific cell populations in vivo by incorporating surface ligands onto vectors that would recognise and bind to distinct receptors on the target [181, 182]. These ligands can take the form of an organic molecule, antibody, aptamer or protein/peptide and permit the vectors to differentiate between healthy tissues and tumour cells [181, 182]. A recent study demonstrated that conjugation of folic acid molecules to polyethylene glycol-succinyl-Chol liposomes facilitated the targeting of the CRISPR/Cas9 vector to ovarian carcinomas where surface folate receptors are expressed in abundance [183, 184]. The increased proximity between the vector and target cell as a consequence of folic acid ligand-folate receptor binding initiates the internalisation of the vector via endocytosis, followed by the release of the vector contents into the cell cytoplasm [184]. In a similar fashion, transferrin ligands can be inserted into the surface of liposomes to target ovarian cancer cells, which also express transferrin receptors in high levels [185]. Additionally, these techniques can be modified to integrate antibodies and peptides such as Angiopep-2 into the synthetic vectors, enabling blood-brain barrier permeation and subsequent gene editing of glioblastoma-associated cells [186–188]. Lastly, cell-based systematic evolution of ligands by exponential enrichment has also generated novel cell-type specific aptamers (single-stranded DNA or RNA oligonucleotides) that could substitute as cell recognition moieties on the vectors targeting osteosarcomeric cells in vivo [189].

## Off-target effects of CRISRP/Cas9 editing in clinically relevant animal models

The repository website, https://clinicaltrials.gov, currently (retrieved August 2, 2019) lists a total of 28 clinical trials where the use of CRISPR/Cas9 technology has been approved in patient treatment. Of the 28 listed trials, 5 have been suspended or withdrawn. In one clinical trial, HIV-positive patients who had developed AIDS and haematological malignancies were infused with allogeneic CD34+ haematopoietic stem/progenitor cells that had the *CCR5* gene ablated by the CRISPR/Cas9 tool to attenuate disease progression [190]. While data from the clinical trial is currently not available, studies have been conducted whereby human CD34+ haematopoietic stem/progenitor cells with *CCR5* gene disruption were infused into immunodeficient mice [191]. The *CCR5* editing efficiency was established to be at around 30%, with a detectable population of edited stem/progenitor cells after 30–47 weeks. These long surviving stem/progenitor cells were able to self-renew and differentiate into multiple cell types of haematopoietic lineage. When exposed to the HIV-1 virus, the mice infused with stem/progenitor cells containing the mutated *CCR5* gene exhibited resistance to HIV-1, as evidenced by the decrease in HIV-1 RNA levels [191]. Careful design of the sgRNA template also minimised OTEs, with whole genome sequencing showing no indels at the closely homologous *CCR2* gene locus and one potential off-target site placed within a nonsense region [191]. While the results were encouraging, detection of OTEs via whole genome sequencing are often limited by the cost associated with high sequencing depth [192, 193]. Hence, it is likely that low frequency OTEs may be missed when the sequencing depth is insufficient (< 10-fold) [194]. To optimise the balance between cost effectiveness and the sensitivity of genome-wide OTEs detection in vivo, a recent study has proposed a new strategy termed “Verification of In Vivo Off-targets” (VIVO) [195]. Briefly, VIVO consists of 2 stages: an in vitro and an in vivo stage. The in vitro stage identifies potential off-target cleavages by CRISPR/Cas9 treatment in vitro via CIRCLE-seq, a next-generation sequencing technique with higher sensitivity for OTEs (due to lower background) than contemporary cell-based detection approaches [196]. The second stage involves the confirmation of off-target sites identified by CIRCLE-seq. Targeted amplicon sequencing was carried out on off-target sites selected for their CIRCLE-seq read counts, in liver tissue harvested from mice treated with viral vectors containing CRISPR/Cas9 components [195]. When the sgRNA targeting the mouse *PCSK9* gene was switched from a less discriminating design to that which were aligned more orthogonally with the mouse genome, no off-target indels (excluding the human *PCSK9* transgene) could be detected by VIVO [195]. The study validated the robustness and sensitivity of VIVO to off-target indels generated by in vivo CRISPR/Cas9 edits, hence presenting a strong claim for its application in clinical therapy [195].

Chimeric antigen receptor (CAR) T cells are T cells genetically engineered to express CARs that contain antigen-recognition and T cell activating domains [197]. CARs facilitate T cell targeting of tumours by binding to specific antigens present on the cancer cell surface and activating cytotoxic pathways to eliminate the cancer cells [198]. In a recently approved clinical trial [199], T cells were transduced with gammaretroviral vectors carrying a CD7 CAR and CD28 endodomain to treat T cell leukaemia/lymphoma [200]. Since T cell lymphomas and non-malignant T cells both express the CD7 glycoprotein, CRISPR/Cas9 gene editing was performed before viral transduction to disrupt the endogenous *CD7* gene and to avoid self-targeting in the CAR T-cells [199]. In vitro studies showed that CD7 CAR T-cells reduced malignant CD7-positive cell lines by at least 95% in cocultures while no observable cytotoxic effect was reported in cocultures with CD7-negative cell lines [200]. Infusing the CD7 CAR T-cells into immunodeficient mice engrafted with tumourigenic CCRF-CEM cells halted the development of leukaemia and prolonged the survival period of the mice. Whole genome sequencing of the CD7 CAR T-cell following CRISPR/Cas9-mediated disruption of the *CD7* gene revealed no significant OTEs when compared to sham controls [200].

Gleaning from the aforementioned complementary preclinical studies, factors such as well-designed, truncated sgRNAs [191] and the delivery of sgRNAs and Cas9 nuclease as an RNP [200] can abate the incidences of OTEs. Furthermore, there are many other in vivo preclinical disease models where rare or non-occurrence of OTEs following CRISPR/Cas9 genome editing lends credibility to the high specificity achievable by this genome editing tool [201–207].

## Conclusion

A well-designed CRISPR/Cas9 study or clinical trial takes into consideration the different aspects in which the gene editing tool can be fully optimised to attain maximal on-target efficiency and minimising OTEs. The absence or rare occurrence of OTEs in preclinical/humanised animal models and clinical pilot studies offers proof of principle that the current level of specificity in CRISPR/Cas9 genome editing permits this technique to be translated onto a larger clinical scale. From a different perspective, the clinician may be forced to choose between administering the gene therapy and incurring the risk of off-target mutations or to forgo therapy and miss the opportunity to diffuse a life-threatening condition. Moreover, if the off-target mutations are non-lethal, will the patient benefit more from gene therapy while coping with the mutation-induced side effects through pharmacological interventions? Lastly, the broad applicability of the CRISPR/Cas9 editing technique demands for strong regulatory institutions and medical ethics boards to prevent any abuse or ethical/moral transgressions, as in the case of Jesse Gelsinger [208] and the *CCR5* gene-edited Chinese babies [209]. Ultimately, the country’s judicial and regulatory bodies have to take into account the political, societal and cultural ideologies and weigh the benefits and risks of gene therapy on a case-by-case basis.

## Abbreviations

ADA^−^SCID: ADA-linked severe combined immuno-deficiency
HR: Homologous recombination
ZFN: Zinc-finger nuclease
TALEN: Transcription activator-like effector nuclease
CRISPR: Clustered regularly interspaced short palindromic repeats
DSB: Double-strand break
NHEJ: Non-homologous end joining
OTE: Off-target effect
pre-crRNA: Precursor CRISPR RNA
tracrRNA: Trans-activating crRNA
PAM: Protospacer adjacent motif
SpCas9: *Streptococcus pyogenes* Cas9
sgRNA: Single-guide RNA
SSB: Single-strand break
dCas9: Deactivated Cas9
indel: Insertion/deletion
UGI: Uracil DNA glycosylase inhibitor
smFRET: Single-molecule Förster resonance energy transfer
SaCas9: *Staphylococcus aureus* Cas9
DMD: Duchenne muscular dystrophy
RNP: Ribonucleoprotein
CMV: Cytomegalovirus
SV40: Simian virus 40
EF1α: Elongation factor 1 alpha
CBA: Chicken β-actin
cTnT: Cardiac troponin T
GFAP: Glial fibrillary acidic protein
miRNA: MicroRNA
Acr: Anti-CRISPR
MRE: miRNA response element
VIVO: Verification of in vivo off-targets
CAR: Chimeric antigen receptor
